# Why parents agree or disagree for minimally invasive tissue sampling (MITS) to identify causes of death in under-five children and stillbirth in North India: a qualitative study

**DOI:** 10.1186/s12887-021-02993-6

**Published:** 2021-11-17

**Authors:** Manoja Kumar Das, Narendra Kumar Arora, Pradeep Debata, Harish Chellani, Reeta Rasaily, Harsha Gaikwad, K. R. Meena, Gurkirat Kaur, Prikanksha Malik, Shipra Joshi, Mahisha Kumari

**Affiliations:** 1 The INCLEN Trust International, New Delhi, 110020 India; 2 Department of Pediatrics, Safdarjung Hospital and Vardhman Mahavir Medical College, New Delhi, 110029 India; 3 Division of Division of Reproductive Biology Maternal and Child Health, Indian Council of Medical Research, New Delhi, 110029 India; 4 Department of Obstetrics and Gynaecology, Safdarjung Hospital and Vardhman Mahavir Medical College, New Delhi, 110029 India

**Keywords:** Minimal invasive tissue sampling, MITS, Counselling, Informed consent, Children, Stillbirth, formative research, qualitative, India

## Abstract

**Background:**

Information on exact causes of death and stillbirth are limited in low and middle income countries. Minimally invasive tissue sampling (MITS) is increasingly practiced in place of autopsy across several settings. A formative research documented the experiences of counselling and consenting for MITS in north India.

**Methods:**

This exploratory qualitative study was conducted at a tertiary care hospital in Delhi. During the early implementation of MITS, observations of the counselling and consenting process (*n* = 13) for under-five child death and stillbirths were conducted. In-depth interviews with MITS team members (*n* = 3) were also conducted. Observation and interview data were transcribed and inductively analysed using thematic content analysis to identify emerging themes and codes.

**Results:**

The MITS team participated in daily ward rounds for familiarisation with parents/families. Following death declaration the counselling was done in counselling corner of the ward or adjacent corridor. Mostly the counselling was targeted at the father and family members present, using verbal explanation and the consent document in local language. The key concerns raised by parents/family were possible disfigurement, time needed and possible benefits. Most of the parents consulted family members before consent. Among those who consented, desire for next pregnancy, previous pregnancy or neonatal loss and participation of treating senior doctor were the key factors. The negative experience of hospital care, poor comprehension and distance from residence were the factors for consent refusal. Lesser number of parents of deceased children consented for MITS compared to the neonates and stillbirths.

**Conclusions:**

The initial experiences of obtaining consent for MITS were encouraging. Consent for MITS may be improved with active involvement of the treating doctors and nurses, better bereavement support, private counselling area along with improvement in quality of care and communication during hospitalisation. Special efforts and refinement in counselling are needed to improve consent for MITS in older children.

**Supplementary Information:**

The online version contains supplementary material available at 10.1186/s12887-021-02993-6.

## Plain English summary

Information about exact cause of death and stillbirth are essential for appropriate care of children and pregnant women. Autopsy assists in establishing exact cause of death, but not preferred by the parents/families. Minimally invasive tissue sampling (MITS) is a suitable alternate to autopsy for establishing causes of death and stillbirth. A pilot project on MITS was initiated at a tertiary care hospital in north India (New Delhi). An exploratory formative research was conducted to document the experiences of counselling and consenting for MITS. Under this study, observation of the counselling and consenting process for conduct of MITS in under-five children (including neonates) and stillbirths were done. Additionally, in-depth interviews with MITS team members were also conducted. Counselling for MITS was done in one corner of the ward or adjacent corridor and mostly targeted at the father and family members present. Counselling was primarily verbal explanation in local language. The key concerns from parents/family were disfigurement, time needed and possible benefits. Most of the parents consulted family members before decision. Benefit during next pregnancy, past previous pregnancy or neonatal loss and senior treating doctor’s involvement were key factors for consent. Poor quality of care and comprehension were the reasons for refusal.

**Supplementary Information:**

The online version contains supplementary material available at 10.1186/s12887-021-02993-6.

## Background

In 2018, 5.3 million under-five child deaths occurred globally, and one-fifth of these children died in India. While half of these deceased children were neonates, about three-fourth were infants [1]. Over 0.5 million stillbirths occur annually in India [2]. India’s National Health Policy (2017) targets to reduce under-five mortality and neonatal mortality rates of 23 and < 10 per 1000 livebirths, respectively by 2025 [3]. The United Nations Sustainable Development Goals (SDG) target achieving under-five mortality and neonatal mortality rates of 25 and 12 per 1000 livebirths, respectively by 2030 [4]. For accelerated decline in child mortality and stillbirths, better information about the causes of death and stillbirth are critical. The current information on causes of death and stillbirth in India and other developing countries are primarily verbal autopsy (VA) based and are unable to assign exact causes especially for the neonates and stillbirths [5–8]. Many of the Indian children die at home and several of those who reach healthcare provider or institutions have limited documentation to assign exact cause(s) of death or stillbirth [9, 10]. The proportion of complete diagnostic autopsy (CDA) conducted in India is dismal due to sociocultural, religious, technical, financial and infrastructure issues [11–15]. In view of the dismal acceptance of CDA, there is need for alternate simpler, feasible and acceptable methods for establishing exact causes of death and stillbirth. In clinical practice, post-mortem body fluid sampling and biopsies are being done to improve diagnosis, but the case selection, methods and samples collected vary across the institutions.

The post-mortem minimally invasive autopsy (MIA) and minimally invasive tissue sampling (MITS) have emerged as a suitable alternative, which include post-mortem examination, imaging (variably) and needle-based tissue sampling from different organs (variably) for histopathologic, microbiological and other desired investigations [16–19]. The MIA and MITS have potential to support establishing accurate cause of death and stillbirth, but are less invasive, non-disfiguring, quicker and cheaper compared to the standard autopsy and feasible in resource limited settings [20–23]. The acceptability for MITS in hypothetical setting has been explored in different socio-cultural, religious and geographic contexts; Asia (India, Pakistan, and Bangladesh), Africa (Ethiopia, Gabon, Kenya, Mali, Mozambique, and South Africa) and Europe (United Kingdom and Belgium) from both parents/families, community and healthcare providers perspectives [24–31]. There was high theoretical MITS acceptability documented in these studies, but also highlighted some challenges related to implementation. Despite the hypothetical acceptance of MITS, there is need for documenting the real-life experiences in obtaining consent for MITS and the contextual factors to guide implementation.

A pilot MITS project under the Child Health and Mortality Prevention Surveillance Network was initiated in north India to document the causes of under-five child deaths and stillbirths. A formative research was undertaken to explore the perceptions, practices, facilitating factors and barriers for MITS. The formative research also attempted to document the process of approaching, counselling to obtain consent for MITS, the questions asked by parents and family, attributes and potential factors influencing the consent. During the early project phase, 44.4% (159/358) of the parents approached gave consent for conducting MITS including 60.2% (50/83) stillborns, 49.2% (92.187) deceased neonates and 20.5% (92/187) deceased children (post-neonatal age). The formative research data were used to understand the factors for acceptance of consent for MITS and suitably inform for refinement in the processes.

## Methods

The formative research conducted during September 2018 to April 2019 had two phases. The Phase-1 focused on exploration of the hypothetical acceptance of MITS from the parents, family, community members and healthcare providers. The Phase-2 aimed to document the process and initial experience of obtaining consent for MITS. The detailed protocol has been published earlier [32]. The manuscripts from formative research Phase-1 findings focusing on acceptance of MITS by parents, community and healthcare providers are under review. This paper presents the findings from Phase-2 of the study.

### Study design and setting

This exploratory qualitative research was conducted at a tertiary care hospital (Safdarjung Hospital) in New Delhi, India. The MITS project focused to document the causes of under-five child death including neonates and stillbirths that occurred at the hospital. The designated MITS team members were trained in sample collection and laboratory procedures. The MITS team included investigators (specialists), paediatricians, neonatologists, obstetricians, pathologists, microbiologists, and research staffs. The research staffs included doctor, nurse-counsellor and technician.

### MITS counselling and consent process

The MITS team coordinated with the pathology and microbiology teams for collection of sample and transfer to laboratory. The MITS team members were trained in counselling techniques and consenting process by the investigators (senior clinicians) through 1 day training followed by hands-on training for 2 weeks and need-based refresher orientations. The MITS team participated in daily ward rounds to identify the critically sick patients and build rapport with the parents/caretakers. In the situations when any child or neonate required end-of-life care, the MITS team was informed by the treating doctor/nurse. The nurse-counsellor provided support to the parents (especially mother) and caretakers present during the process. The deaths and stillbirths that occurred during the daytime only were targeted for MITS, due to logistic feasibility. Once the death was declared by the treating doctor, the resident doctor introduced the MITS team to the parent/caretaker present in the hospital. The MITS team (doctor and nurse-counsellor) approached, requested and moved the parents/caretakers/responsible family members to the designated area in the wards or corridor outside the ward to ensure privacy for counselling. The MITS team briefly discussed with the parent’s and/or family member’s understanding about the cause of the death or stillbirth and family context (other children). Then, they informed about the MITS procedures and emphasized on the method including sampling and needle biopsy process, non-disfigurement, time needed, availability of reports and possible benefits. They encouraged the parent/family members to ask question and concerns. When feasible, the senior investigators and consultants participated in the consenting process. The study information sheet in local language (Hindi) and verbal explanation were used. No visual or pictorial tool were used for counselling and consenting process. Once the consent document was signed, the body was moved to the MITS room. Following the MITS procedure, the body was covered appropriately and returned to the ward. The nurse completed the documentation and handed over the body to the family. If parent/family wanted to see the body after MITS, the body was shown before handover.

### Study participants

The participants included parents and family members of deceased children and stillbirths, the MITS research staff and healthcare providers, who were present at the hospital during counselling. In-depth interviews with the MITS team members (*n* = 3) were conducted. For the observations, the parents and family members approached by the MITS team were included (*n* = 13).

### Data collection

The data collection involved observation of the processes of interactions between the parents/family members and MITS research team while explaining for consent and during handover of the body for those who consented for MITS. Verbal consent for observation and documentation of the MITS counselling and consent process was obtained from the parent or family member who was approached for MITS. A pair of female researchers (GK, PM, MK, and SJ, qualification PhD or MPH) trained in qualitative research observed the process and took notes of the verbal, non-verbal expressions by the parents/family members and MITS research staff and healthcare providers and flow of events (Supplementary file-1). The observation was conducted in the ward and/or outside ward area, where the counselling were done. In view of the sensitivity of the situation and process, no interview with parents/family or audio-recording was done for these observations. IDIs with the MITS team members were done in separate room (in the hospital) in local language using the interview guide (Supplementary file-2) and audio recorded with consent. During the interviews no person besides the participant and researchers (two) was present. The counselling for obtaining consent for MITS by MITS team took about 30 (20–45) minutes and interviews by the qualitative research team lasted for 45–60 min. The lead author (MKD, male paediatrician with over 15 year experience in qualitative research) led and supervised the data collection.

### Data handling and analysis

The field notes taken by the research team member during the observations and interviews were transcribed verbatim in local language using a time-event-activity sequence matrix. The observation and interview transcripts were translated into English. The data entered into computer were saved into the server and backed up on daily basis. The transcripts were read by two researchers independently several times for analysis. The inductive data analysis followed the grounded theory and phenomenology methodological principles. The steps followed during analysis were: free listing, domain identification, coding, and cross tabulation. The emerging codes and themes were discussed periodically to resolve the discrepancies. These emerged codes were reviewed and grouped into the axial codes and then selective codes and assembled under key themes. A reiterative process was adopted for analysis, coding and thematic summarization.

### Ethical considerations

The observations of MITS counselling were done after obtaining verbal consent from the parent/family member involved in the MITS consent. The in-depth interviews were done after obtaining written informed consent. The confidentiality and anonymity of participants were assured. The study protocol was reviewed and approved by the institute ethics committees of the participating institutes (The INCLEN Trust International and Safdarjung Hospital).

## Results

### Observation of counselling and consenting process

Till April 2019, MITS team approached parents/family members of 27 deaths or stillbirths for consent. The cases approached for consent included five stillbirths, 16 neonates and six children (post-neonatal age). Out of these approached cases, consent from parents was obtained for 12 cases including four stillbirths, six neonates and two child deaths. The formative research team observed the counseling and consenting process in thirteen cases including one stillbirth, seven newborns and five child deaths. Table 1 summarizes the characteristics of these thirteen cases. Out of these observed cases, consent for MITS being performed was obtained in five cases including one stillbirth, three neonates and one child. As reflected in Table 1, in eight cases some additional family members were present with parents or father during the counselling. In one case although both parents were present, the maternal uncle discussed about the consent.

**Table 1:** Characteristics of the cases approached to obtain consent for minimally invasive tissue sampling (MITS) and consent status

	Age category	Period of hospitali-sation	Place of death	Diagnosis	Members present	Consent for MITS
Case 1	< 1 month	1 day	Ward	Preterm, RDS and shock	Father, grandmother	Yes
Case 2	> 1 month- < 1 year	3 days	Ward	Downs syndrome, pneumonia and sepsis	Both parents, Grandmother, and two male members	Yes
Case 3	< 1 month	2 days	Nursery	Preterm and septic shock	Both parents and two male members	No
Case 4	> 1–5 years	5 days	Ward	CHD, pneumonia and septic shock	Both parents and one male member	No
Case 5	> 1 month- < 1 year	0 day (7 h)	Ward	LBW, NEC and septic shock	Father and one male member	No
Case 6	> 1 month- < 1 year	2 days	NICU	LBW, pneumonia and septic shock	Mother, Grandfather and one male member	No
Case 7	> 1–5 years	1.5 day	Ward	AGE with dehydration and septic shock	Both parents	No
Case 8	> 1–5 years	2.5 days	Ward	Pneumonia and septic shock	Both parents and grandparents	No
Case 9	< 1 month	1 day	Ward	Birth asphyxia and shock	Father and grandparents	No
Case 10	< 1 month	2 days	Nursery	Preterm and congenital pneumonia	Both parents	No
Case 11	< 1 month	1 day	Nursery	Preterm and VLBW	Father	Yes
Case 12	< 1 month	2 days	Nursery	Birth asphyxia and septic shock	Father	Yes
Case 13	–	–	Labour room	Stillbirth	Father	Yes

Abbreviations: *AGE* Acute gastroenteritis, *CHD* Congenital heart disease, *LBW* Low birth weight, *MITS* Minimally invasive tissue sampling, *NEC* Necrotizing enterocolitis, *NICU* Neonatal intensive care unit, *RDS* Respiratory distress syndrome, *VLBW* Very low birth weight

#### Process of counselling and conduct of MITS

In most observed cases, both the MITS doctor and nurse-counsellor were involved in counselling for MITS. The team looked for the father and/or male family member to approach and counsel them. During counselling, they explained about the need for knowing the cause of death or stillbirth and potential benefits for them and for care of other children or pregnant women. The team also emphasized the procedure and less-invasive nature of MITS, use of needle-based sampling and availability of reports. In three cases the neonatology consultants involved in patient care also participated in the counselling process and consent was obtained in two cases. The consent document was signed by the father. When both parents were present, they consulted between themselves and in other cases father consulted with the family members physically present or over phone before agreeing for MITS. For one deceased neonate, the father initially denied for MITS. But the grandmother pushed and father finally agreed for MITS. Usually the counselling process and decision for consent took about 25–45 min. The MITS procedure took about 60–120 min. The parents and family members waited in the ward or near the MITS room during the MITS process.

#### Questions asked by family and explanations

The key issues raised by the families were chances of disfigurement, chance of blood oozing from pricked sites, organ removal, time needed, necessity and possible benefits. The key concern raised by all parents/family was body disfigurement. Several parents/families asked questions “*Will you do post-mortem?”; “Will you do cutting of the child?”; “Will you do operation?”* and several parents wanted to see the body after MITS. Some parents/family asked about the time to receive the reports. For some parents/family they desired to move quickly out of the hospital and refused consent. Data saturation was achieved by twelfth case of approach to consent.

*“Will all diseases be known, will everything be known? There should not be any cutting of the baby, all be taken through injection only. (Father of stillborn, Observation 10, Consented)*

*“It means you will take the child and do some cutting. You can take the samples by needle here (in the ward) also.” (Father of deceased newborn, Observation 12, Consented)*

#### Reactions, emotions and interactions

All parents expressed emotional outbursts in the form of crying in variable degrees. Before death declaration, the mothers were moved outside the ward and they returned to the ward once the demise news was conveyed to them by one of the family members. It took about 15–20 min before the parent or family member could be approached for explaining about MITS and consent. The parents of three cases were not receptive while explanation and later refused saying that the child has already died and will not return with the tests. Few parents wanted one of them to be present in the MITS room. The MITS team explained patiently about the process and replied to the questions as needed.

#### Decision making process

The decision making process appeared to be collective in majority of the cases. The father although consented and signed the document, he consulted the other family members present with him or consulted over phone before making decision. Some consent refusals were also finalised after consultation with family members. In one case the maternal uncle discussed and decided although the parents were available.

#### Factors for acceptance and refusal

Out of the five cases undergone MITS, three were preterm newborns, one child with Down syndrome (with history of stillbirth to the parents) and one stillbirth. Thus, the parents/families who consented explained they were moved by the potential value of the MITS and possible benefit for the next pregnancy. It was also observed that more parents of male deceased/stillborn child consented for MITS.

*“He (her son) has two daughters. This child was a boy, but died. Will the test help in knowing what to do for the next pregnancy?”*

*(Grandmother of deceased newborn, Observation 1, Consented)*

*“I am agreeing to give consent, so that this does not happen again. When so many tests have been done, it is ok to do one more.”*

*(Father of deceased child with Down syndrome, Observation 2, Consented)*

For the parents/families who refused consent, the common factors that influenced decision were dissatisfaction with the care and services received and no additional value perceived. Some families from outside the state and deaths that occurred towards later part of the day desired to return home quickly for completing burial rituals on same day. It also appeared that obtaining consent in child deaths was more challenging than the neonates and stillbirths. In one case, although the father appeared willing, but later refused after discussion with family.

*“Whatever was bound to happen has happened, we are not finding it (MITS) right”.*

*(Grandfather of deceased child, Observation 6, Not consented)*

*“No, now we don’t want to do any test. I was upset for so long for my baby, when you were supposed to do these tests then you did not do anything. Now what can you do, there is no use as my baby is no more”. (Father of deceased newborn, Observation 5, Not consented)*

*“Whatever you want to do, do it in the ward and you can take 10 minutes. After that I will go. I have arranged the vehicle and we have to go far.”*

*(Father of deceased child, Observation 7, Not consented)*

#### Age of the child

We observed that lesser number of parents of children consented for MITS than the neonates and stillbirths.

#### Role of treating doctors

Some of the parents wanted to discuss with the treating senior doctor for decision making. In four instances the consultant also counselled and three of them consented for MITS.

#### Experience of the MITS team

The MITS team faced challenges in the initial period for mobilising and coordinating with the treating resident doctors and nurses in the ward for transfer of the body and return to ward for handover to parents. The team members were less confident about the process of explaining and obtaining consent for MITS and their confidence gradually improved after 10–15 sessions. According to them participation of the consultants helped in improving the consent acceptance. They felt that the involvement of the resident doctors and nurses in the wards in the counselling could have improved the consent acceptance for MITS.

## Discussion

This study documented the process of counselling and consenting for MITS at a tertiary care hospital in India. The MITS team approached the parents/family quickly after death declaration for seeking consent. During counselling, the parents/family had key concerns about the procedure, possible disfigurement, chance of blood oozing, organ removal, procedure time and time needed for reports availability. The decision making was collective and made in consultation with elders and family. The factors for MITS acceptance were: Desire for the next healthy pregnancy, deceased/stillborn being male and mother having suffered previous bad pregnancy outcomes. For consent refusal, non-satisfactory experience of hospital care and perceived adequacy or benefit of information about the cause of death were key factors apart from the perceived value of MITS, time constraints and desire to return home quickly and age of the child. We observed that lesser number of parents of deceased children consented for MITS compared to the deceased neonates and stillbirths. The involvement of senior doctors appeared to improve consent acceptance.

The processes of counselling and obtaining consent were similar to the experiences at five sites across Asia (India, Pakistan and Bangladesh) and Africa (Kenya and Ethiopia) conducting MITS [33]. According to the report, different types of personnel were involved in the counselling and consenting process; doctors, midwives and nurses in Pakistan; resident doctors in South India; social scientist/psychologist in Bangladesh; psychologist and nurses in Kenya and resident doctors and nurses in Ethiopia who had undergone training variably. In Pakistan and Bangladesh, the counsellors participated in the clinical rounds for rapport building with families, as done at our site. In Ethiopia, the counsellors first counselled for complete diagnostic autopsy (CDA) and then offered MITS, if parents refused for CDA. The counselling was done mostly in the wards/corridor or open space outside the wards or in separate rooms. All sites used primarily verbal method for counselling. To answer the religious concerns, Quran quotes in Pakistan and Ethiopia and Fatwa from religious body in Bangladesh were used. While treating physicians/nurses were directly involved in South India and Ethiopia, their assistance was sought as needed in Pakistan and Bangladesh. The MITS teams who were not part of the treating team faced challenges in consenting. The other challenges encountered were timing of death (those occurred in night), parent/family education level (those with low education were unable to understand), religious factors (Muslims), and opinion of extended family and mother’s health status (for stillbirths). Several findings from our observations were comparable to similar findings reported in other studies, especially from South India, Pakistan and Bangladesh [33]. These findings include families approached with 1 hour after death, involvement of expended family in consent process, time taken for decision, MITS perceived to be less-invasive and non-disfiguring than the CDA, perceived potential benefit by parents with past stillbirths and/or neonatal deaths and influence of treating physician’s participation on counselling.

Another study from Kenya (where post-mortem was conducted for child deaths) reported that the parents/family previously consented had higher education and better knowledge about autopsy. Majority who consented wanted to know the cause of death and possible future benefit. Those who refused consent perceived no necessity of post-mortem, but majority of them would have accepted MITS, if offered then [34]. High hypothetical acceptance of MIA/MITS have been reported from various countries in Asia, Africa and Europe [25, 30, 31, 35, 36].

The post-mortem rates have progressively declined globally. A study from United Kingdom identified multidisciplinary involvement and involvement of senior staff in counselling as critical factors for consent to conduct post-mortem [37]. Another study from United Kingdom found positive response (89% of the approached) for autopsy by changing the mode of communication, which was contrary to the healthcare provider perceptions. The key concerns raised during consent were body disfigurement and delays in funeral [38]. A report from United States highlighted that 63% of parents of deceased children were not offered autopsy and majority of them would have considered, if it had been offered [39]. In a survey, parents who consented for post-mortem after child death or pregnancy loss wanted to know the cause (44%) and improving medical knowledge (24%). Majority of them felt benefited knowing the cause (60%) and possible implication for future pregnancy (21%). Few respondents did not find post-mortem to be useful due to confusing information, complex language, inadequate communication and additional visits. The main reasons for not consenting were, child suffered enough (44%), perceived no benefit (26%) and disfigurement (10%). Few of those who didn’t consent also regretted for not agreeing [36].

The proportion (44.4%) of parents/family consented for MITS in this study was similar to the observations from Ethiopia (39.8%), but lesser than South Africa (65.7% for neonatal deaths and 65.5% for post-neonatal deaths) [23, 40, 41]. The lower proportion of consent among the post-neonatal deaths was a challenge. The factors including attachment with the child, sociocultural practices and treating physician’s/nurse’s involvement in counselling could have influenced the consent for MITS.

These observations indicate that multiple factors influence acceptance of autopsy and MITS in different sociocultural contexts including the parental/family factors (desire to know cause, educational level, religion, family composition, past experiences, extended family influence, residence and time of death), healthcare provider related factors (perceived benefit, perceived consent acceptance, quality of care, communication, rapport with parents and counselling quality) and institutional factors (infrastructural support, multidisciplinary and enabling system). The perceived benefit for the next pregnancy and bad pregnancy outcome in past were the possible reasons of higher MITS acceptance among neonatal deaths and stillbirths. Presence of other live children and higher emotional attachment with the deceased child (fear of disfigurement and pain) were the probable reasons for higher refusal by parents of deceased children. Despite the high hypothetical acceptance of MITS, there were challenges in real-life context and documentation is needed for informing ongoing and future implementation.

Independent observation of the counselling, consenting, MITS and post-MITS procedures were strengths of our study. The small sample size is a limitation. The findings reflect practices at one hospital, which may be context specific and hence may not be generalizable. The results could have been influenced by the parent’s characteristics. No interview was conducted with parents/family.

## Conclusions

This study described the process of counselling and obtaining consent for MITS in North Indian hospital context. The initial experiences of obtaining consent for MITS were encouraging in view of the overall acceptance. The consent for MITS among child deaths was lower than the neonates and stillbirths, which needs further exploration and efforts. The consent acceptance can be furthered with active engagement of the treating doctors and caring nurses, better bereavement support, dedicated and private counselling area accommodating the extended family and overall effort for improving the care and communication during the hospitalisation period. The future experiences with obtaining consent to conduct MITS in stillbirth, neonates and older children from different sociocultural contexts are needed to expand the technique to other centres.

## Supplementary Information

**Additional file 1.**

**Additional file 2.**

## Data Availability

The datasets used and analyzed during the current study are available from the corresponding author on reasonable request.

## Abbreviations

CDA: Complete diagnostic autopsy
MIA: Minimally invasive autopsy
MITS: Minimally invasive tissue sampling
VA: Verbal autopsy
