# Supplementary material for: Why parents agree or disagree for minimally invasive tissue sampling (MITS) to identify causes of death in under-five children and stillbirth in North India: a qualitative study
Source: BMC Pediatr. 2021 Nov 17;21:513. doi: 10.1186/s12887-021-02993-6 (PMC8597286; doi:10.1186/s12887-021-02993-6)
Supplement: Supplementary file 1 — Additional file 1. [file 12887_2021_2993_MOESM1_ESM.pdf]

Study Title: A pilot study to determine causes of death in under-five children in a tertiary hospital in India using the MITS technique: A feasibility and acceptability study (Sociobehavioral Study Component)

Observation of the process of obtaining consent for MITS for the child (including neonates) deaths and stillbirths occurred in the hospital

Date of observation (Date):

Ward/Unit:

Information about the deceased:
Name: Age: Sex:

Date of death (date):

Date of hospitalization (date):

Cause of death (as declared by the doctor):

Family members present:

Counseling done by:

Treating doctor present:

Treating nurse present:

Observation start time (HH:MM): Observation end time (HH:MM):

	Narration of the discussion
1	Initial briefing and explanations given by the MITS team

	Narration of the discussion
2	Questions asked by the parents or family members<br>*(Please note who asked the question and record the statement as mentioned.)*<br><br>Response given by the MITS team<br>*(Please note who responded and record the statement as mentioned.)*
3	Questions asked by the parents or family members<br>*(Please note who asked the question and record the statement as mentioned.)*<br><br>Response given by the MITS team<br>*(Please note who responded and record the statement as mentioned.)*
4	Questions asked by the parents or family members<br>*(Please note who asked the question and record the statement as mentioned.)*<br><br>Response given by the MITS team<br>*(Please note who responded and record the statement as mentioned.)*
5	Questions asked by the parents or family members<br>*(Please note who asked the question and record the statement as mentioned.)*<br><br>Response given by the MITS team<br>*(Please note who responded and record the statement as mentioned.)*

	Narration of the discussion
8	Questions asked by the parents or family members<br>*(Please note who asked the question and record the statement as mentioned.)*<br><br>Response given by the MITS team<br>*(Please note who responded and record the statement as mentioned.)*
7	Questions asked by the parents or family members<br>*(Please note who asked the question and record the statement as mentioned.)*<br><br>Response given by the MITS team<br>*(Please note who responded and record the statement as mentioned.)*
8	Any additional family member joined/invited/consulted by the parent/family member?<br>*(If yes, please mention the relationship, mode of consultation.)*
9	Any doctor/nurse from the treating team joined/invited/consulted by the MITS team?<br>*(If yes, please mention the counseling/ explanation given.)*
10	Decision on consent for MITS by the parent/family.<br><br>If refused, the reason(s) mentioned by them.

Any other specific observations

Observing team members Signature
