# Supplementary material for: Why parents agree or disagree for minimally invasive tissue sampling (MITS) to identify causes of death in under-five children and stillbirth in North India: a qualitative study
Source: BMC Pediatr. 2021 Nov 17;21:513. doi: 10.1186/s12887-021-02993-6 (PMC8597286; doi:10.1186/s12887-021-02993-6)
Supplement: Supplementary file 2 — Additional file 2. [file 12887_2021_2993_MOESM2_ESM.pdf]

Study Title: A pilot study to determine causes of death in under-five children in a tertiary hospital in India using the MITS technique: A feasibility and acceptability study (Sociobehavioral Study Component)

In-depth interview guide- MITS Team member

1. **Basic demography of the respondent**
   1.1. Designation
   1.2. Gender
   1.3. Educational qualification
   1.4. Total years of service
   1.5. Total period in current position

Service delivery and communication

2. Who are the MITS team members and what are their roles and responsibilities.
   Please describe your role related to the MITS activities.

3. Please narrate the processes regarding approaching the parents/family members for consent to conduct MITS?
   Probe: Who is approaches and when (time after death)? Where are they approached?

4. What all items do you use for explaining the parents/family members for obtaining consent?
   Probe: Materials/tools used or demonstrated.

5. Are there different strategies adopted by you and your team while approaching the parents of stillborn, deceased nenoates and children?

6. How do you and the MITS team members build rapport with the parents and family members of the patients?
   Probe: Who engages with the parents and when?

7. What have been your experience with the process of obtaining consent for MITS till now?
   Probe: Success rate and questions asked by them.

8. Some parents/family members have consent for MITS. In your opinion, why parents/family members are agreeing for MITS?

9. Some parents/family members have refused consent for MITS. In your opinion, what are the reasons for refusal by them?

10. What are the important challenges faced by you and your team? How have you overcome the challenges?

11. What all have helped/assisted you and your team in obtain consent for MITS?
    Probe: Ask for details about these, persons, processes, etc.

12. How do the treating doctors and consultants/specialists assist you in the process of obtaining consent for MITS?

13. How do the nurses you in the process of obtaining consent for MITS?

14. How have you been trained on the processes and counseling to obtaining consent for MITS?
    Probe: Place, duration, Trainer/facilitator, hands-on support

15. What are the challenges faced by you for conducting MITS?

16. Can you please narrate some important lessons and instances that you remember while obtaining consent for MITS.

17. Any other comment/ suggestions.

Thank the respondent for his/her critical contribution.
